# Supplementary material for: Effects of HIV Infection in Plasma Free Fatty Acid Profiles among People with Non-Alcoholic Fatty Liver Disease
Source: J Clin Med. 2022 Jul 2;11(13):3842. doi: 10.3390/jcm11133842 (PMC9267237; doi:10.3390/jcm11133842)
Supplement: Supplementary file 1 [file jcm-11-03842-s001.zip › jcm-1784611-supplementary.pdf]

Effects of HIV infection in plasma free fatty acid profiles among people with non-alcoholic fatty liver disease

Supplementary Materials

Table S1. Antiretroviral regimens used by HIV-positive participants (n = 31)

Drug regimen	N (%)
**INSTI-based**	
ABC + 3TC + DTG	12 (39)
TDF + FTC + EVG/c	10 (32)
TDF + 3TC + DTG	2 (6)
3TC + DTG	2 (6)
TDF + FTC + DTG	1 (3)
ABC + 3TC + RAL	1 (3)
RPV + DTG	1 (3)
**NNRTI-based**	
ABC + 3TC + NVP	1 (3)
**PI-based**	
RPV + DRV/c	1 (3)

Abbreviations: 3TC, lamivudine; ABC, abacavir; DTG, dolutegravir; EVG/c, cobicistat-boosted elvitegravir; FTC, emtricitabine; INSTI, integrase strand transfer inhibitor; NNRTI, non-nucleoside reverse transcriptase inhibitor; NVP, nevirapine; PI, protease inhibitor; RAL, raltegravir; RPV, rilpivirine; TDF, tenofovir disoproxil fumarate

Table S2. Adjusted differences in plasma individual fatty acids from NAFLD patients by HIV status

	**Coefficient**	**95% CI**	**p-value**
Lauric (C12:0)	0.00	-0.65, 0.65	0.994
iso-tetradecanoic (C14:0 *iso*)	-1.58	-3.78, 0.62	0.151
Myristic (C14:0)	0.45	-0.03, 0.94	**0.065**
Pentadecanoic (C15:0)	1.72	-0.21, 3.64	0.078
iso-hexadecanoic (C16:0 *iso*)	-0.92	-2.86, 1.02	0.339
Palmitic (C16:0)	0.17	-0.08, 0.11	0.716
anteiso-heptadecanoic (C17:0 *anteiso)*	-0.23	-1.88, 1.42	0.778
Margaric (C17:0)	-0.45	-2.85, 1.95	0.703
Stearic (C18:0)	-0.10	-0.32, 0.11	0.328
Arachidic (C20:0)	0.04	-0.83, 0.91	0.925
Behenic (C22:0)	1.57	0.36, 2.79	**0.013**
Lignoceric (C24:0)	-0.67	-1.11, -0.23	**0.004**
Myristoleic (C14:1 *cis*9)	-13.01	-28.12, 2.11	**0.089**
Palmitoleic (C16:1 *cis*9)	-0.01	-0.27, 0.24	0.907
trans-palmitoleic (C16:1 *trans*)	5.25	0.22, 10.29	**0.041**
Heptadecenoic (C17:1)	1.13	-0.76, 3.02	0.231
Oleic (C18:1 *cis*9)	0.16	-0.03, 0.05	0.414
Cis-vaccenic (C18:1 *cis*11)	-0.16	-0.71, 0.38	0.546
Rumenic (C18:2, *cis*9, *trans*11)	0.83	-0.96, 2.63	0.348
Linoleic (C18:2)	-0.01	-0.04, 0.03	0.625
Dihomo-gamma-linolenic (C20:3)	-0.19	-0.59, 0.20	0.328
Arachidonic (C20:4)	-0.03	-0.12, 0.07	0.547
Alpha linolenic (α C18:3)	1.22	0.10, 2.34	**0.033**
Eicosapentaenoic (C20:5)	0.17	-0.40, 0.75	0.540
Docosapentaenoic (C22:5)	0.00	-1.60, 1.60	0.999
Docosahexaenoic (C22:6)	0.06	0.29, 0.41	0.715
Dimethyl acetal C16 (DMA C16:0)	0.60	-0.52, 1.72	0.282
Dimethyl acetal C18 (DMA C18:0)	-0.58	-1.49, 0.32	0.198

Analyses adjusted for sex, body mass index, and alcohol and dairy consumption. Coefficient indicates the adjusted difference in fatty acid concentration of HIV-infected participants compared to HIV-uninfected participants (reference category).

Table S3. Adjusted differences in fatty acid groups and calculated ratios estimating desaturases and elongases activity, according to HIV status

	Coefficient	95% CI	p-value
**ΣSFA**	0.00	-0.09, 0.09	0.931
**ΣMUFA**	0.13	-0.23, 0.05	0.458
**ΣPUFA**	-0.01	-0.04, 0.02	0.546
**Σomega-3**	0.12	-0.13, 0.37	0.338
**Σomega-6**	-0.01	-0.04, 0.02	0.461
**SFA/PUFA**	0.31	-0.76, 1.39	0.557
**SFA/MUFA**	-0.10	-0.89, 0.69	0.791
**MUFA/PUFA**	0.35	-0.40, 1.11	0.342
**Omega-6/omega-3**	-0.01	-0.04, 0.01	0.307
**Desaturases**			
Delta-5 omega-3 (C20:5/C18:4)	-0.04	-0.15, 0.07	0.478
Delta-5 omega-6 (C20:4/C20:3)	0.00	-0.09, 0.09	0.927
Delta-6 omega-3 (C22:6/C22:5)	0.01	-0.02, 0.04	0.626
Delta-9 (C14:1/C14:0)	-8.04	-17.39, 1.30	**0.089**
**Elongases**			
C16:0/C14:0	-0.02	-0.04, 0.00	**0.052**
C18:0/C16:0	-1.32	-4.89, 2.25	0.456
C20:0/C18:0	1.11	-5.27, 7.49	0.724
C22:0/C20:0	0.35	-0.10, 0.80	0.122
C24:0/C22:0	-0.06	-0.10, -0.01	**0.018**
C20:3/C18:3	-2.35	-12.40, 7.71	0.636
C18:1/C16:1	0.01	-0.02, 0.03	0.641
C22:5/C20:5	-0.22	-0.57, 0.14	0.220

Analyses adjusted for sex, body mass index, and alcohol and dairy consumption. Coefficient indicates the adjusted difference in plasma concentrations among HIV-infected participants compared to HIV-uninfected participants (reference category)

Figure S1. Observation plot representing the fatty acids on the dimensions extracted from the principal component analysis.

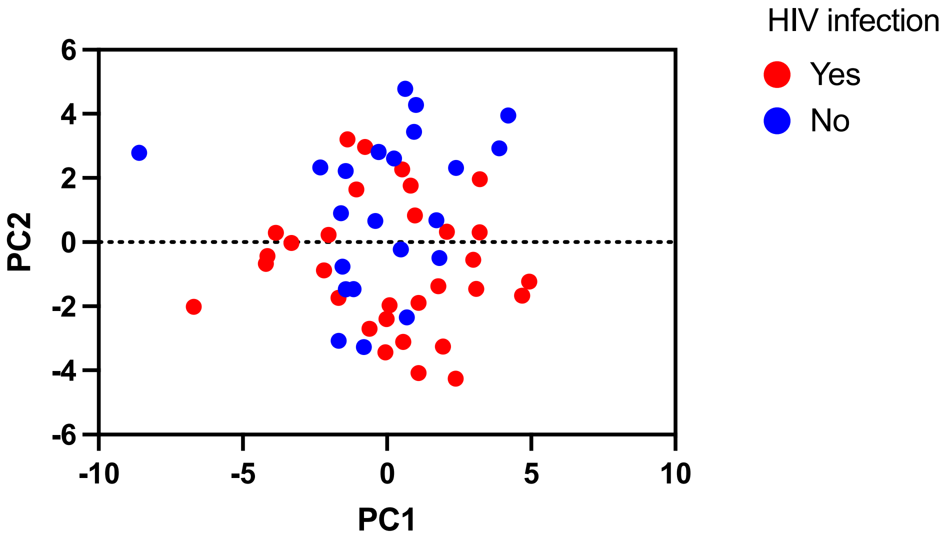

Explained variance: PC1 = 15%; PC2 = 12%

PC1 and PC2 represent the two dimensions that explain the greatest amount of variance.
